# Feasibility and Effectiveness of an Urgent Care–Community Partnership to Reduce Disparities in Patient Portal Uptake: Quality Improvement Project

**DOI:** 10.2196/69253

**Published:** 2025-06-05

**Authors:** Mechelle Sanders, Amaya Sanders, Erik Herbert, Naomi Rosie Booker, Sandy Wang, Kevin Fiscella

**Affiliations:** 1 Department of Family Medicine, University of Rochester, 1381 South Ave, Rochester, NY, 14620, United States, 1 5853244566, 1 5854732245; 2 College of Arts and Sciences, Howard University, Washington, DC, United States; 3 Wegmans School of Pharmacy, St. John Fisher University, Rochester, NY, United States; 4 School of Global Public Health, New York University, New York, NY, United States; 5 Emergency Medicine, Urgent Care, Department of Family Medicine, University of Rochester, Rochester, NY, United States

**Keywords:** patient portal, urgent care, digital divide, navigation, equity, disparities

## Abstract

**Background:**

Patient portals demonstrate significant potential for improving health care engagement but face critical adoption challenges. Disparities persist across different demographic groups, creating a digital divide in health care access. Targeted training strategies, particularly personalized and one-on-one approaches, show promise in increasing portal utilization. Innovative solutions, like community health workers specializing in digital navigation, offer a potential pathway to reduce enrollment barriers. The key challenge remains developing a scalable, cost-effective training model.

**Objective:**

Our quality improvement (QI) project aimed to assess the feasibility and effectiveness of a collaborative effort between a free community-based digital navigation program and an urgent care clinic in facilitating patient access to their portal.

**Methods:**

We created the Digital Health Equity Navigation Training (DHENT) program to improve patient portal access and usage. The program used a train-the-trainer model to scale up patient portal training across the community. DHENT trainers partnered with urgent care physicians to enroll patients in the portal. Physicians briefly explained portal benefits and referred interested patients for DHENT assistance. Trainers then contacted patients by phone to help with enrollment and navigation. We employed 3 Plan-Do-Study-Act cycles to understand the feasibility of the collaboration. We used descriptive statistics to describe participant characteristics and referral processes.

**Results:**

The collaboration was marginally successful, exceeding referral targets by 27.7% (115/90). Most patients were under 60 years old (94/115, 81.7%) and White (78/115, 67.8%). There was a significant delay in contact, averaging 37 days. While 4.8% (5/104) of patients accessed the portal with DHENT trainer assistance, 9.6% (10/104) had already signed up independently after their urgent care visit.

**Conclusions:**

Overall, we found our partnership had a moderate impact, and only a low dose of intervention and resources were needed.

## Introduction

### Problem

Physicians in one of the University of Rochester’s urgent care clinics identified the need to increase patient portal access and use in their practice. They believed portal use would be beneficial for reducing time spent outside the clinical encounter, making patients aware of nonemergent updates to their health information that was readily available in their patient portal (eg, lab results). The problem was that they did not have a systematic way to remind patients to sign up, flag, or support those who needed help accessing their portal outside the clinical encounter.

### Available Knowledge

A systematic review found patient portal interventions to be overall effective in improving medication adherence, some psychological outcomes, and preventive service use [1]. Varady et al [2] determined that portal use was independently associated with lower no-show rates, which they estimated corresponded to US $218,225 in yearly savings for their health system. Unfortunately, disparities in patient portal use persist by sex, age, morbidity, and health literacy [3].

Patient training can address nonuse. One-on-one interventions have the most evidence for increasing portal use in vulnerable populations [4]. However, training can vary in how it is delivered (eg, live or in person, via videos) and by whom it is delivered (eg, physician, nurse, navigator). In a randomized controlled trial, in-person patient portal training delivered by a trained study team member for hospitalized patients led to increased portal use and improved patient satisfaction and engagement. Patients who received personalized training accessed the portal more often and used more portal functions compared to those who only watched training videos [5]. Digital navigators (DNs) are a potentially cost-saving, individually delivered training strategy that shows promise for reducing patient portal disparities. DNs are lay professionals, like community health workers, who tend to work closely with the health care system and focus on patients’ use of digital health tools while addressing barriers such as digital literacy. A pilot DN program designed to reduce racial and ethnic disparities in patient portal uptake in a primary care setting increased portal enrollment among Black and Hispanic patients who had low enrollment rates prior to the program [6].

### Rationale

The optimal training approach remains unclear, both in terms of who the trainers should be and how to implement collaborative training strategies. Research has revealed differences in portal uptake based on who engaged them about it. One study found disparities in portal utilization patterns between patients trained by residents versus attending primary care providers, with residents’ patients demonstrating lower engagement [7]. These findings have significant implications for intervention delivery costs. For example, the time an attending physician dedicates to training a patient may be nonbillable and detract from other patient care. Conversely, DNs may offer a more cost-effective alternative, but patient uptake may be lower, thereby negating any cost savings.

A hybrid training approach between physicians and lower-cost trainers may therefore be best. As the race to close the digital divide in patient portal use persists, a comprehensive evaluation of factors influencing adoption and long-term sustainability is crucial [7].

### Specific Aims

Our quality improvement (QI) project aimed to assess the feasibility and effectiveness of a collaborative effort between a free community-based DN program and an urgent care clinic in facilitating patient access to their portal.

## Methods

### Context

The University of Rochester Department of Family Medicine and Health Equity Program Support Office partnered with local community leaders to create a Digital Health Equity Navigation Training (DHENT) program. The goal of the program was to improve access and use of the health system’s patient portal (MyChart), increase the community’s awareness of no or low-cost internet services, and gather data on the community’s digital health needs.

DHENT employed a train-the-trainer model, offering free training to individuals who agreed to train others within their respective communities and organizations that provided direct care (especially community health workers, peer navigators, promotores, etc). This approach allows for a more efficient and scalable way to implement patient portal training across larger communities. Among our initial trainees were 3 undergraduate students and a Public Health AmeriCorps Service Member summer volunteer.

### Intervention

The DHENT curriculum was originally designed for working with patients face-to-face. We later tailored it to be appropriate for the telephone navigation [6]. For example, rather than using the “show-me” method (“Can you show me how you would find your recent lab test results?”), the trainers asked patients to explain in detail what they saw on their screens and used verbal cues to confirm the patient’s progress through each step (“Tell me what words, shapes, or colors you see on your screen”). We conducted two 90-minute training sessions with the trainers. We charged them with three primary goals for their patients: (1) educate them on the benefits of MyChart for their care, (2) identify and overcome any barriers to accessing MyChart (eg, recovering an email password or linking them to free resources in the community), and (3) help them navigate key functions within the portal on their own.

The DHENT trainers partnered with an urgent care practice within our health care system to support 6 physicians in enrolling their patients in the portal. DHENT trainers and urgent care physicians were not colocated. At the end of an urgent care visit for adult patients who were not actively using the portal, each physician agreed to spend 2‐3 minutes explaining the benefits of the portal and encouraging the patient to enroll. When warranted, physicians asked patients for consent to be contacted by DHENT via telephone for further assistance with sign-up.

### Participants

**Figure 1.**
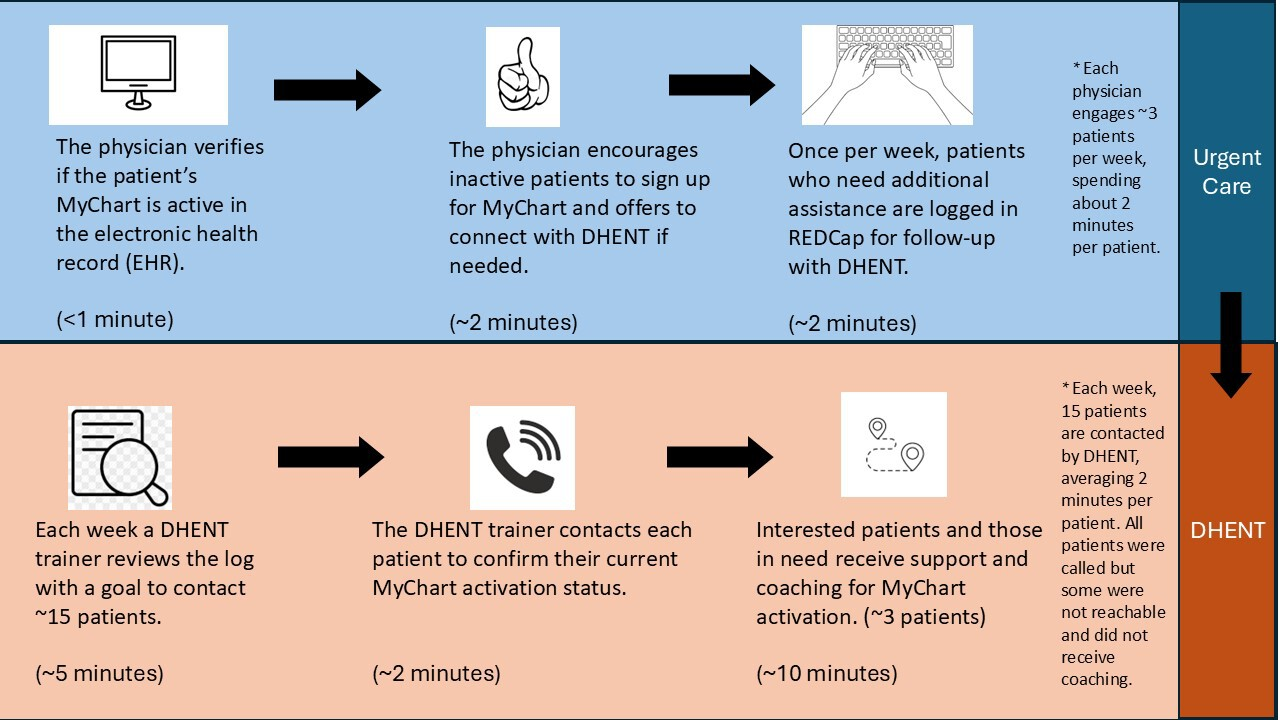
Flow diagram illustrating the weekly process for identifying and logging patients who require additional assistance for follow-up with DHENT. DHENT: Digital Health Equity Navigation Training; REDCap: Research Electronic Data Capture.

Six physicians were trained by a practice champion (an urgent care physician) during a team meeting. The practice champion told them about the purpose of the project and demonstrated how to refer patients. Specifically, the physicians learned how to complete a brief 8-item survey in Research Electronic Data Capture (REDCap; a secure and web-based platform for data collection) to (1) provide the contact information for each patient they referred (eg, name; phone number; email address, if available) and (2) inform the DHENT team about any known barriers the patient had to signing up (eg, requests for an access code) (Figure 1).

### Measures and Analysis

We used descriptive statistics to describe participant characteristics and referral processes. Feasibility was based on reach (number of referred patients divided by number of anticipated referrals per physician), time (length of time between physician referral and DHENT trainer contact with the patient), and participation rates (number of referred patients per number of contacted patients). Effectiveness was defined as the percent of patients who accessed their portal during the phone call.

### Ethical Considerations

This project was undertaken as a quality improvement (QI) initiative and, according to the University of Rochester’s Guideline for Determining Human Subject Research (Human Subject Research Determination Checklist) [8], did not meet the definition of human participant research as outlined in the US Health & Human Services Common Rule 45 CFR 46 [9]. No compensation was provided to participants. Data were shared on a secure database accessible only to the study team, and patients provided verbal consent to clinicians prior to inclusion. Because the project did not meet the definition of human participant research, formal written informed consent was not required.

### Study of the Intervention

We employed 3 Plan-Do-Study-Act (PDSA) cycles to understand the feasibility of the collaboration.

#### PDSA Cycle 1

The goal was for each physician to engage 3 patients per week and log them in the REDCap database. For the first 2 weeks, only 2 physicians had logged 8 patients. The practice champion determined that some of the physicians had engaged patients but did not have time to log them in REDCap. Given that, the practice champion volunteered to offer support to those who needed help with data entry and entered their data at the end of each week. As a result, the DHENT trainers reviewed the updated list once per week and made phone calls.

#### PDSA Cycle 2

The practice champion checked in with the DHENT trainers each week to assess emergent needs. The team added a data field to REDCap so a physician could indicate when a patient spoke a language other than English or was deaf or hard of hearing. By the end of week 4, five physicians had logged 38 patients.

#### PDSA Cycle 3

Continuity of care with urgent care patients and their physicians is challenging because there is no long-term patient-provider relationship. This makes it difficult to verify information (such as phone numbers) during future visits. This, combined with lagged data entry and DHENT contact, left many patients unreachable by the DHENT team. At the end of cycle 3, the phone-based DHENT support ended. However, the physicians continued to remind patients to sign up for their portal and provided brief in-house support (eg, resetting access codes or verifying login information) to patients at the end of the visit.

## Results

The program was piloted with a sample of 125 adult patients who visited the urgent care practice from May 2024 to July 2024. DHENT trainers made phone calls to patients one day per week from June 2024 to August 2024. The trainers completed a brief survey after they attempted to contact each patient. Questions included the outcome of the attempt (eg, unable to contact, helped a patient sign up, left a message) and any open-ended notes about their experience during the telephone encounter.

The collaboration was feasible with marginal success. We exceeded our target number of referrals by 27.7% (115/90). Of the 125 patients who were engaged by their urgent care physician, 115 were then referred to DHENT, and 104 had complete contact information. Physician referrals ranged from 1 to 46 patients. The average time between referral and DHENT contact (including at least 2 attempts via voice message) was 37 days (median 43, range 16-70 days). Most patients were less than 60 years old (94/115, 81.7%) and White (78/115, 67.8%).

DHENT trainers were unable to speak to 51.9% (54/104) of the patients and left them a voicemail message. They were unable to contact 17.3% (18/104) due to a wrong phone number, the phone not being in service, or an inability to leave a voicemail message. While 4.8% (5/104) of patients accessed the portal with the DHENT trainers, 9.6% (10/104) had already signed up on their own since leaving their urgent care appointment (Table 1). Finally, 1.9% (2/104) of patients told the DHENT trainer they were no longer interested in accessing their portal.

**Table 1.** Digital Health Equity Navigation Training (DHENT) trainers phone call outcomes.

DHENT phone call outcomes	Value (n=104), n (%)
Left patient a message	54 (51.9)
Assisted patient in accessing MyChart	5 (4.8)
Provided patient with MyChart education	1 (1.0)
Patient unwilling to sign-up for MyChart	2 (1.9)
Patient unable to sign-up for MyChart	2 (1.9)
Patient’s phone not in service	4 (3.8)
Rescheduled (patient currently unavailable)	10 (9.6)
Wrong phone number	5 (4.8)
Voicemail box full, unable to leave patient a message	8 (7.7)
Patient already signed-up for MyChart	11 (10.6)
Patient hung up the phone	2 (1.9)

## Discussion

### Summary

Our DHENT trainers were unable to contact more than half of the patients that were referred to the program. However, for those that were contacted, they were able to leverage physician endorsement and DHENT trainer experience to engage patients. We found large variation in referrals per physician. We are unsure if this indicates problems with the referral process for some physicians or if there needs to be more done to increase physician interest and awareness of the program.

A few patients enrolled in their portal on their own before they were contacted by DHENT. This may mean that not all the patients that were identified by the urgent care physicians genuinely needed help. Better strategies for identifying patients in need can reduce resource inefficiency and divert DHENT time to those who truly need it. However, we cannot discount the possibility that some patients may have reported they enrolled in the portal but did not actually do so. The DHENT trainers were unable to validate the patients’ self-report. Second, there was a significant lag between physician referral and first contact. DHENT trainers only made telephone calls once per week. This low-dose intervention and the delay in contacting patients may have reduced their interest in accessing their portal. Our findings align with those of Rodriguez et al [6], which show that DNs struggle to reach and enroll all patients that are referred to digital navigation services. Nonetheless, their rates were still higher than ours; they reached 74% of their referrals compared to 48% for DHENT. However, it is important to note their program had more resources. Their navigator was employed and colocated, worked closely with the health care team, and sent information to patients via postal mail about the portal. The demographics of patients in our study differed from what we anticipated. Our sample was predominantly White and somewhat younger than the populations typically reported in previous studies as less likely to enroll in the patient portal (i.e., individuals aged 65 years and older). [3,10,11]. This may signal sampling bias but may also underscore the impact of the location of the urgent care centers and demographics of patients that are most likely to use them [12,13].

### Lessons and Limitations

The findings from this QI project have important implications for future practice and research in health interventions. The potential for scalability through partnerships with volunteer programs such as DHENT presents an opportunity to extend the reach of digital support for patients. Undergraduate students receive real-world patient experience to support future endeavors, and patients receive support. This model serves as a viable framework for health care practices with limited or no resources. Overall, our findings underscore the importance of community involvement, teamwork, and resourcefulness in developing effective interventions for patients.

Patient portals are becoming an increasingly used communication tool. Patients unable to access them may face significant barriers to digitally engaging their care unless efforts are made to provide support from alternative means outside the clinical encounter. Our project highlights the need for more robust evidence to show whether low-cost or low-resource approaches such as volunteer phone outreach can be better tailored to meet patient needs over more resource-intensive approaches such as face-to-face, point-of-care interventions. Although less robust, approaches such as this may better align with resource availability in safety-net practices and with the preferences and time availability of patients and their families.

Despite the successful implementation of the partnership, there were some notable limitations. First, this was designed as a QI project. Future studies should rigorously test our approach and its impact on patient health-related outcomes. Second, we did not collect any information on patient or physician satisfaction. These types of information are necessary for understanding long-term sustainability. Future studies should look to include a more diverse and representative sample of patients, thereby enhancing the applicability of our findings.

### Conclusions

Overall, we found our partnership had a marginal impact, and only a low dose of intervention and resources was needed.

## Abbreviations

DHENT: Digital Health Equity Navigation Training
DN: digital navigator
PDSA: Plan-Do-Study-Act
QI: quality improvement
REDCap: Research Electronic Data Capture
